# Resveratrol Treatment Is Associated with Lipid Regulation and Inhibition of Lipoprotein-Associated Phospholipase A2 (Lp-PLA2) in Rabbits Fed a High-Fat Diet

**DOI:** 10.1155/2020/9641582

**Published:** 2020-05-20

**Authors:** Lei Xu, Renjie Wang, Hongyu Liu, Jiaoqi Wang, Jing Mang, Zhongxin Xu

**Affiliations:** ^1^Department of Neurology, China-Japan Union Hospital of Jilin University, Changchun City, Jilin Province 130033, China; ^2^Department of Nuclear Medicine, China-Japan Union Hospital of Jilin University, Changchun City, Jilin Province 130033, China

## Abstract

The effects of resveratrol on various conditions have been widely studied previously. This paper aimed to investigate the influence of resveratrol on atherosclerosis (AS). Twenty-four New Zealand male rabbits were randomly and equally assigned to the normal diet group (NDG), fat diet group (FDG), and fat diet with resveratrol group (80 mg/kg/d, RFG). Biochemical indicators from blood samples were analyzed at baseline and 3 months to investigate the effects of resveratrol on blood lipid, lipoprotein-associated phospholipase A2 (Lp-PLA2), liver, and renal function. The indicators including alanine aminotransferase (ALT), aspartate aminotransferase (AST), creatinine (CREA), triglycerides (TG), total cholesterol (TC), high-density lipoprotein cholesterol (HDL-C), low-density lipoprotein cholesterol (LDL-C), and Lp-PLA2. At 3 months, arteries were stained with hematoxylin and eosin to study the influence of resveratrol on the aortic intima, smooth muscle layer, and the intima/media ratio. Comparisons of weight, ALT, AST, CREA, TG, TC, HDL-C, LDL-C, and Lp-PLA2 among the three groups showed no significant difference at baseline. However, at the end of 3 months, significant differences were observed in AST, CREA, TC, HDL-C, LDL-C, and Lp-PLA2 between the three groups (*P* < 0.05). In pairwise comparison, CREA, TC, LDL-C, and Lp-PLA2 had significant differences between any two groups (*P* < 0.05). In addition, there were significant differences in the AST and HDL-C levels between RFG and NDG groups (*P* < 0.05). Meanwhile, the HDL-C levels were also significantly different between the FDG and NDG groups (*P* < 0.01). The histologic analysis also showed that the thickness of the aortic intima and the ratio of the intima and aortic tunica media (*P* < 0.05) significantly decreased in RFG compared to FDG. Resveratrol may have an antiatherosclerosis effect on a rabbit model of AS.

## 1. Introduction

Atherosclerosis (AS) is a disease that seriously endangers human health. Numerous basic and clinical studies indicate that AS is a chronical inflammatory disease, characterized by an abnormal reaction to the damage of blood vessel walls, with symptoms including classic inflammatory degeneration, exudation, and proliferation characteristics [1]. AS can induce a variety of fatal ischemic cardio-cerebrovascular diseases, such as cerebrovascular disease, coronary heart disease, and thromboembolic disease [2]. With the increase in life expectancy caused by improved living standards, more attention has been put on the damage caused by cardiovascular and cerebrovascular diseases [3].

Resveratrol is a polyphenolic compound that can be naturally produced by numerous plants, including grapes, peanuts, and mulberries [4]. There are different opinions about the effect of resveratrol on atherosclerosis. A study by Wilson showed that resveratrol promotes atherosclerotic development, rather than protecting against it, by a mechanism that is independent of observed differences in gross animal health, liver function, plasma cholesterol concentrations, or low-density lipoprotein (LDL) oxidative status [5]. However, numerous studies both in vitro and in vivo have shown that resveratrol can protect against atherosclerotic cardiovascular disease through several potential mechanisms, including mitigation of inflammation [6] or oxidative stress [7], regulation of energy metabolism [8], enhancement of endothelial function and vasorelaxation [9], and inhibition of platelet aggregation [10]. In vitro cell culture studies have also shown that while inhibiting foam cell formation, resveratrol can also improve the metabolism of lipoproteins and reverse the transportation of cholesterol [11, 12].

However, it is still controversial whether resveratrol can improve AS and alter blood lipids and function of liver and kidney. There are also limited data concerning the effect of resveratrol on blood lipoprotein-associated phospholipase A2 (Lp-PLA2). Lp-PLA2, also known as platelet-activating factor acetylhydrolase (PAF-AH), is a 45 kD, calcium-independent enzyme which “belongs to the phospholipase A2 superfamily. Lp-PLA2 can be produced by inflammatory cells in atherosclerotic plaques, such as monocytes, macrophages, T-lymphocytes, and mast cells [13, 14]. In blood circulation, under the effect of chemotactic inflammatory cells, Lp-PLA2 generates a self-reinforcing positive feedback cycle, generating proinflammatory substances and leading to the occurrence and development of atherosclerosis [15, 16]. A study by Sun revealed that resveratrol caused a decrease in macrophage Lp-PLA2 levels and reduced the infiltration of inflammatory cells in the mouse liver [17]. A study by Luo showed that resveratrol assumed a protective position adjacent to the phospholipid head group of the phospholipid bilayers, proximal to fatty acyl chains, and was able to protect phospholipids from hydrolytic attack by PLAs [18]. However, the effect of resveratrol on Lp-PLA2 levels in an AS model remains to be investigated. Due to the important role of Lp-PLA2 in the development of AS, it is necessary to further study the regulation of resveratrol on Lp-PLA2 in AS. The activities of AS, an inflammatory disease, can be assessed by measuring the levels of circulating biomarkers. Sufficient evidence has been accumulated for Lp-PLA2 to be used as a biomarker in clinical practice [19, 20]. With a high specificity for vascular inflammation and a direct role in the causal pathway of plaque inflammation, Lp-PLA2 is relatively unique in its high specificity, making it an exemplary biomarker [21–23].

Two main animal AS models have been developed for the study of the molecular mechanisms underlying AS: one is induced by a high-fat diet and another involves intimal injury [10, 24]. Due to the experimental limitations of the intimal injury method, caused by restrictions of the equipment, the fat diet is considered to more accurately follow the chronic progression of human AS, although it requires more time. Thus, in the present study, we utilized the fat diet rabbit AS model to investigate the influence of resveratrol on blood lipids, Lp-PLA2, liver/kidney function, and AS.

## 2. Materials and Methods

### 2.1. Ethical Statement

All experiments were approved by the Institutional Animal Care and Use Committee at the China-Japan Union Hospital of Jilin University. All procedures were performed in accordance with the National Institute of Health's Guide for the Care and Use of Laboratory Animals.

### 2.2. Rabbits

Three-month-old New Zealand male white rabbits, weighing approximately 2 kg, were obtained from Changchun Yis Laboratory Animal Technology (Jilin, China). The rabbits were given free access to food and water, while housed under a natural day/night cycle.

**Figure 1:**
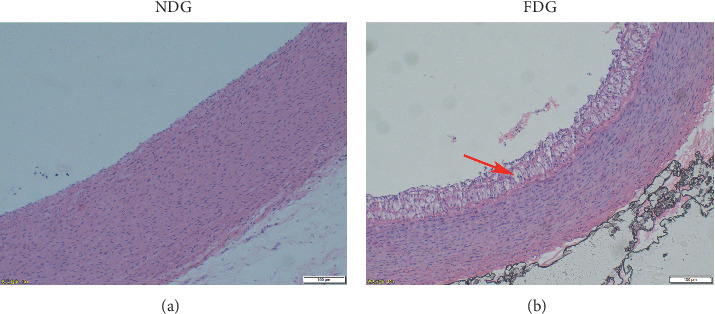
Representative photomicrographs of aortic arch sections stained with hematoxylin-eosin of a normal diet group (NDG) and a fat diet group (FDG) in the preliminary experiment. Magnification: 10x. Bar represents 100 *μ*m. The red arrow indicates that, compared with the NDG, foam cells formed under the intima in the FDG. Meanwhile, the intima was thickened, and plaques were formed. This showed that the atherosclerosis model was successfully established after a high-fat diet.

To test the hypothesis that the fat diet can induce AS, a preliminary experiment was conducted. A control (NDG) rabbit was fed on a normal diet (NDG), while an experimental (FDG) rabbit fed was fed on a fat diet. The normal diet contained carbohydrates 68%, protein 22%, salt 0.5%, and fat 10%, and the fat diet contained 1% cholesterol, 5% lard, 5% yolk powder, and 89% basic rabbit feed (Beijing Botai Hongda Biotechnology Co., Ltd., China). The rabbits were maintained on this diet for 3 months. The aortic arch was harvested from the rabbits after euthanasia by injection with sodium phenobarbital (0.2 g/kg, diluted concentration 3.5%) into the ear vein and put in a test tube containing 4% paraformaldehyde for histological evaluation. Perpendicular to the vascular axis, the aortic arch was cut into approximately 2 mm lengths, embedded in paraffin and stained with hematoxylin and eosin. Atherosclerotic lesions were detected by light microscopy in the rabbit fed on a fat diet. This model has previously been used for studies of AS [25, 26]. As shown in Figure 1, foam cells were formed under the intima, the intima was thickened, and plaques were formed in the FDG group rabbit. This preliminary experiment offered evidence that the high-cholesterol diet was capable of causing atherosclerosis.

Twenty-four rabbits were randomly assigned to three groups: a normal diet group (NDG) served as a control; a fat diet group (FDG); and an FDG group treated with resveratrol (RFG). The treatment concentrations of resveratrol were determined according to a preliminary experiment and a literature review (Beijing Solarbio Science & Technology Co., Ltd., China). Resveratrol (80 mg/kg/day) was mixed in with the feed (50 g, twice a day) and accounted for about 0.2% of the feed [17, 27].

### 2.3. Blood Chemistry

Blood samples were collected by heart puncture from the rabbits in the three groups at the baseline, before commencement of the diet in the morning, and after 3 months. The determinations of blood levels of alanine aminotransferase (ALT, IU/L), aspartate aminotransferase (AST, IU/L), creatinine (CREA, µmmol/L), triglycerides (TG, mmol/L), total cholesterol (TC; mmol/L), high-density lipoprotein cholesterol (HDL-C, mmol/L), low-density lipoprotein cholesterol (LDL-C, mmol/L), and Lp-PLA2 (diluted 10-fold, ng/mL) were conducted. A chemiluminescent methodology was used to analyze the levels of ALT, AST, CREA, TG, TC, HDL-C, and LDL-C on the AU5800 autoanalyzer (Beckman Coulter, USA) via serum detection kits (Beckman Coulter, USA). Dry-type fluorescence immunity analysis (Guang Zhou Labsim Biotech Co., Ltd., China) was performed to determinate the levels of Lp-PLA2 using a lipoprotein-related phospholipase A2 kit (Vazyme Biotech Co., Ltd., China), and the methodology was quantum dot fluorescence immunoassay.

### 2.4. Histological Analysis

After euthanasia of the rabbits by injection with sodium phenobarbital (0.2 g/kg, diluted to a concentration of 3.5%) into the ear vein, perpendicular to the vascular axis, the aortic arch was cut into approximately 2 mm lengths and placed in a test tube containing 4% paraformaldehyde. Samples were embedded in paraffin and stained with hematoxylin and eosin. Measurements of the thickness of the intima and smooth muscle layer were conducted by light microscopy (Olympus Corporation, Japan). The software connected with the microscope screenshot has a measurement tool. Clicking the measure button, the measured value will automatically appeared with dragging from a certain point to zero. After measuring the thickness of the intima and the smooth muscle layer under the microscope, the mean and standard deviations were determined, and the intima/middle layer ratio was subsequently calculated.

### 2.5. Statistical Analysis

All statistical analyses were performed using the SPSS 19.0 software (IBM, Armonk, New York); data from the experiments were analyzed and expressed as the mean ± standard errors of the means (SEM). One-way analysis of variance (One way ANOVA) was used for further analysis. If the variance between groups was homogeneous, a comparison was conducted using the Bonferroni method, and the *P* value was corrected. Dunnett's test method was adopted for cases of uneven variance.

## 3. Results

### 3.1. Laboratory Examinations

The comparison of weight, ALT, AST, CREA, TG, TC, HDL-C, LDL-C, and Lp-PLA2 levels between the three groups showed no significant difference at the baseline. The differences in pairwise comparison between the three groups also showed no significant difference at the baseline (*P* < 0.05) (Table 1).

Intergroup comparison at the end of the 3-month experiment showed that the levels of AST, CREA, TC, HDL-C, LDL-C, and Lp-PLA2 were significantly different among the three groups (*P* < 0.05), as shown in Table 2. In a pairwise comparison, CREA, TC, LDL-C, and Lp-PLA2 showed a significant difference between any two groups (*P* < 0.05). Additionally, AST (77.91 ± 24.54 vs 40.51 ± 6.46) and HDL-C (1.58 ± 0.35 vs 1.03 ± 0.20) were significantly different between RFG and NDG (*P* < 0.05). Meanwhile, HDL-C (3.28 ± 1.61 vs 1.03 ± 0.20) was also significantly different between the FDG and NDG groups (*P* < 0.05). No statistical significance in any other component was found with the pairwise comparison.

In addition, intragroup blood index comparisons were performed in the NDG, FDG, and RFG rabbits between the two time points: baseline and at 3 months. There were no significant differences in ALT, TG, LDL-C, and Lp-PLA2 levels between the baseline and after 3 months in NDG rabbits (Table 3). Meanwhile, the level of TG showed no significant difference in the FDG rabbits, and no significant difference in ALT in FDG and RFG was seen over the course of the experiment (Table 3).

### 3.2. Histologic Analysis

**Figure 2:**
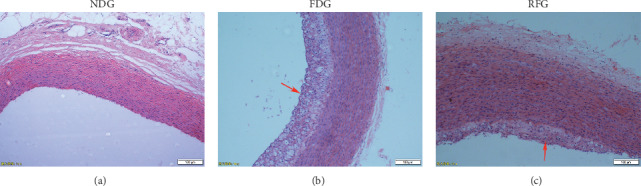
Representative photomicrographs of aortic arch sections stained with hematoxylin-eosin of a normal diet group (NDG), a fat diet group (FDG), and a resveratrol-treated FDG group (RFG). Magnification: 10x. Bar represents 100 *μ*m. The red arrows indicate the thickening intima and plaque formation in the FDG and RFG, which were compared with the NDG. The intimal thickness of RGF was significantly lower than that of FDG (*P* < 0.05).

Figure 2 shows that the intima thickness of the aorta after RFG treatment was significantly decreased when compared with FDG, as was the ratio of the intima and aortic tunica media. The smooth muscle layers of the aortic arch in the three groups had no significant difference (Table 4).

## 4. Discussion

The prevention and treatment of cardiovascular and cerebrovascular diseases is an important topic of medical research, and studies have shown that AS participates in the development of many of these diseases [28]. In the present study, a stable rabbit AS model was established through 3 months of fat diet feeding. Utilizing this rabbit model, the relationship between resveratrol and AS was analyzed. Our results showed that the thickness of the intima and the ratio of the intima/media were significantly different in the RFG and FDG. Meanwhile, the value of CREA, TC, LDL-C, and Lp-PLA2 were significantly different between any two groups. Previous studies have shown that the formation of AS was accompanied by the proliferation of vascular endothelial cells and smooth muscle cells, among others [29]. By reducing the aortic intima area and the intima/media ratio, resveratrol inhibited the progression of atherosclerotic lesions [30]. In a study of the same rabbit model, resveratrol prevented the development of atherosclerotic lesions through the reduction of invasion of foam cells in the tunica media, [31]. In the present study, the thickness of the intima and the intima/media ratio were reduced by the intervention of resveratrol, which showed a significant difference between RFG and FDG. The thickness of the smooth muscle layer was also reduced by resveratrol intervention, but the difference between RFG and FDG was not significant.

Some studies had found that Lp-PLA2 was associated with plaque progression and vulnerability [32, 33]. In our study, Lp-PLA2 was significantly different in the RFG (953.20 ± 96.66) and FDG (1928.88 ± 385.78) groups when compared with the NDG (520.14 ± 51.55) group (*P* < 0.05). Meanwhile, with resveratrol intervention, Lp-PLA2 levels in the RFG were significantly lower than those in the FDG (*P* < 0.05). In circulating blood, Lp-PLA2 binds to lipoproteins through apolipoprotein (Apo) B, thereby hydrolyzing oxidized phospholipids in ox-LDL to produce lipid proinflammatory substances such as lysolecithin and oxidized free fatty acids [34]. These lipid proinflammatory substances can cause vascular endothelial cell death and endothelial dysfunction, stimulate the production of adhesion factors and cytokines, and produce atherosclerosis [16]. It is possible that resveratrol increases the expression of superoxide dismutase, catalase and glutathione peroxidase, and other antioxidant enzymes, thereby reducing the formation of free radicals, preventing endothelial damage, and directly blocking the inner membrane of blood vessels. In addition, resveratrol inhibits the production of proinflammatory cytokines and further reduces the atherosclerotic effect of Lp-PLA2 [35]. At the same time, resveratrol can inhibit the absorption of low-density lipoproteins by macrophages, prevent low-density lipoprotein peroxidation, inhibit lipid peroxidation, and regulate levels of blood lipids [36].

Lipid disorders are closely associated with the development of AS [37]. Studies have attributed the retention of serum lipoproteins in the artery wall to AS [38]. A cascade of chronic proinflammatory events in the artery wall were initiated by the accumulation of oxidized lipoproteins. The chronic inflammation observed in AS was caused by the recruitment of macrophages and the uptake of lipids into these cells [39]. According to previous studies, reduction of TC and LDL-C levels in patients with coronary heart disease could significantly reduce the mortality and recurrence rate of cardiovascular events [40, 41]. In the present study, intervention with resveratrol significantly decreased the levels of TC and LDL-C in rabbits fed a high-fat diet. The mechanism by which resveratrol inhibits the formation of advanced atherosclerotic lesions appears to be through its inhibitory effects on LDL oxidation [42]. Resveratrol has been shown to protect lipids from peroxidative degradation and to stop the uptake of oxidized LDLs in the vascular wall in a concentration-dependent manner [43, 44]. Thus, we hypothesized that resveratrol affected AS through impact on lipid TC and LDL-C levels.

AST and ALT enzymes are mainly concentrated in the liver and are involved in the conversion of sugars and proteins in vivo [45]. With the cell membrane fragmentation and permeability increase induced by liver cell damage, ALT and AST are activated and released into the blood. When liver cells are damaged, cells undergo degeneration, necrosis, and cell membrane fragmentation or permeability increase [46]. At this time, the ALT and AST contained in liver cells is released into the blood, thus increasing ALT and AST activity in the blood. Currently, AST and ALT are commonly used to detect liver injury in a clinic [37]. In the present study, a decrease in AST levels was detected in the comparison between RFG and FDG; however, this difference was not significant.

CREA is a product of human muscle metabolism, which is produced by the irreversible nonenzymatic dehydration of creatine [47]. The concentration of CREA in serum increased with the functional impairment of the kidney and is therefore a reliable indicator of renal function [48]. Studies have shown that lipid metabolism disorder is a factor in the development of chronic kidney disease, and resultant kidney damage can aggravate the dysregulation of lipid metabolism [49]. CREA has been widely used as a common test for kidney injury [50, 51]. In our study, CREA values increased with resveratrol treatment in rabbits fed a high-fat diet (*P* < 0.05). Resveratrol has previously been reported to cause nephrotoxicity [52]. The possible reason behind this is that the kidneys are the dominant excretion organ, and the recovery rate of total resveratrol in the urine and feces fluctuates between 70% and 98% within 24 hours. Therefore, in our study, compared with the FDG, the CREA values were significantly increased in RFG (*P* < 0.05).

In summary, the present study suggests that resveratrol could inhibit the incrassation of arterial intima. In addition, resveratrol treatment reduced the levels of TC, LDL-C, Lp-PLA2, and CREA in rabbits fed a high-fat diet. Thus, resveratrol may have a function in preventing AS. However, clinical trials are needed to further investigate this effect.

## 5. Conclusions

Resveratrol might have antiatherosclerosis effect on a rabbit model of AS.

## Figures and Tables

**Table 1:** Biochemical analysis of rabbits from NDG (*n* = 8), FDG (*n* = 8), and RFG (*n* = 8) analyzed before the beginning of the fat diet (baseline).

		NDG (*n* = 8)	FDG (*n* = 8)	RFG (*n* = 8)	*F*	*P* value
Weight (kg)		0.96 ± 0.05	0.99 ± 0.08	1.01 ± 0.08	0.903	0.420
ALT		43.20 ± 9.65	41.22 ± 8.83	44.76 ± 12.80	0.226	0.800
AST		36.38 ± 8.61	33.73 ± 5.91	34.88 ± 4.98	0.317	0.732
CREA		67.94 ± 8.39	64.72 ± 6.27	65.75 ± 9.69	0.318	0.731
TG		0.63 ± 0.20	0.50 ± 0.16	0.48 ± 0.07	2.230	0.132
TC		1.94 ± 0.21	1.88 ± 0.28	1.99 ± 0.43	0.240	0.789
HDL-C		0.93 ± 0.19	0.94 ± 0.25	0.93 ± 0.17	0.006	0.994
LDL-C		0.84 ± 0.22	0.95 ± 0.12	0.80 ± 0.27	1.053	0.367
Lp-PLA2^#^		461.50 ± 89.57	525.48 ± 32.50	494.88 ± 32.56	2.424	0.113

^#^The test result of homogeneity of variance was *P* < 0.05, and Dunnett's T method was used for multiple comparison of *P* value correction, while the Bonferroni method was used for the other indicators. NDG, normal diet group; FDG, fat diet group; RFG, fat diet with resveratrol; ALT, alanine aminotransferase; AST, aspartate aminotransferase; CREA, creatinine; TG, triglycerides; TC, total cholesterol; HDL-C, high-density lipoprotein cholesterol; LDL-C, low-density lipoprotein cholesterol; Lp-PLA2, lipoprotein-associated phospholipase A2.

**Table 2:** Biochemical analysis of rabbits from NDG (*n* = 8), FDG (*n* = 8), and RFG (*n* = 8) analyzed at the end of the 3-month fat diet.

	NDG (*n* = 8)	FDG (*n* = 8)	RFG (*n* = 8)	*F*	*P* value
Weight (kg)^#^	2.90 ± 0.08	2.98 ± 0.15	2.96 ± 0.07	1.160	0.333
ALT	44.81 ± 7.95	50.86 ± 24.53	61.06 ± 20.26	1.506	0.245
AST^#^	40.51 ± 6.46	84.32 ± 54.94	77.91 ± 24.54^a^	3.668	0.043
CREA	77.38 ± 10.45	111.92 ± 11.38^a^	127.63 ± 10.84^a,b^	44.526	<0.001
TG	0.62 ± 0.21	0.90 ± 0.42	0.76 ± 0.28	1.567	0.232
TC^#^	2.19 ± 0.30	30.32 ± 5.74^a^	11.84 ± 2.78^a,b^	120.264	<0.001
HDL^#^	1.03 ± 0.20	3.28 ± 1.61^a^	1.58 ± 0.35^a^	11.946	<0.001
LDL	0.91 ± 0.22	16.45 ± 3.16^a^	6.23 ± 1.53^a,b^	120.880	<0.001
Lp-PLA2^#^	520.14 ± 51.55	1928.88 ± 385.78^a^	953.20 ± 96.66^a,b^	77.698	<0.001

^#^The test result of homogeneity of variance was *P* < 0.05, and Dunnett's T method was used for multiple comparison of *P* value correction, while the Bonferroni method was used for the other indicators. NDG, normal diet group; FDG, fat diet group; RFG, fat diet with resveratrol; ALT, alanine aminotransferase; AST, aspartate aminotransferase; CREA, creatinine; TG, triglycerides; TC, total cholesterol; HDL-C, high-density lipoprotein cholesterol; LDL-C, low-density lipoprotein cholesterol; Lp-PLA2, lipoprotein-associated phospholipase A2. ^a^Statistically significant when compared to NDG at *P* < 0.05. ^b^Statistically significant when compared to FDG at *P* < 0.05.

**Table 3:** Biochemical analysis of rabbits from RFG (*n* = 8) before the beginning of the treatment with fat diet and at the end of the 3-month fat diet.

	Baseline (*n* = 8)	After 3 months (*n* = 8)	*t*	*P* value
Weight(kg) (NDG)	0.96 ± 0.05	2.90 ± 0.08	−73.655	<0.001
Weight (kg) (FDG)	0.99 ± 0.08	2.98 ± 0.15	−49.925	<0.001
Weight (kg) (RFG)	1.01 ± 0.08	2.96 ± 0.07	−39.000	<0.001
ALT (NDG)	43.20 ± 9.65	44.81 ± 7.95	−0.781	0.461
ALT (FDG)	41.22 ± 8.83	50.86 ± 24.53	−1.172	0.280
ALT (RFG)	44.76 ± 12.80	61.06 ± 20.26	−2.160	0.068
AST (NDG)	36.38 ± 8.61	40.51 ± 6.46	−2.490	0.042
AST (FDG)	33.73 ± 5.91	84.32 ± 54.94	−2.639	0.033
AST (RFG)	34.88 ± 4.98	77.91 ± 24.54	−5.587	0.001
CREA (NDG)	67.94 ± 8.39	77.38 ± 10.45	−7.002	<0.001
CREA (FDG)	64.72 ± 6.27	111.92 ± 11.38	−10.794	<0.001
CREA (RFG)	65.75 ± 9.69	127.63 ± 10.84	−12.390	<0.001
TG (NDG)	0.63 ± 0.20	0.62 ± 0.21	0.086	0.934
TG (FDG)	0.50 ± 0.16	0.90 ± 0.42	−2.230	0.061
TG (RFG)	0.48 ± 0.07	0.76 ± 0.28	−2.986	0.020
TC (NDG)	1.94 ± 0.21	2.19 ± 0.30	−2.919	0.022
TC (FDG)	1.88 ± 0.28	30.32 ± 5.74	−14.078	<0.001
TC (RFG)	1.99 ± 0.43	11.84 ± 2.78	−9.275	<0.001
HDL (NDG)	0.93 ± 0.19	1.03 ± 0.20	−3.359	0.012
HDL (FDG)	0.94 ± 0.25	3.28 ± 1.61	−3.815	0.007
HDL (RFG)	0.93 ± 0.17	1.58 ± 0.35	−4.880	0.002
LDL (NDG)	0.84 ± 0.22	0.91 ± 0.22	−1.270	0.245
LDL (FDG)	0.95 ± 0.12	16.45 ± 3.16	−13.489	<0.001
LDL (RFG)	0.80 ± 0.27	6.23 ± 1.53	−9.221	<0.001
Lp-PLA2^#^ (NDG)	461.50 ± 89.57	520.14 ± 51.55	−1.930	0.095
Lp-PLA2^#^ (FDG)	525.48 ± 32.50	1928.88 ± 385.78	−10.090	<0.001
Lp-PLA2^#^ (RFG)	494.88 ± 32.56	953.20 ± 96.66	−12.816	<0.001

^#^The test result of homogeneity of variance was *P* < 0.05, and Dunnett's T method was used for multiple comparison of *P* value correction, while the Bonferroni method was used for the other indicators. NDG, normal diet group; FDG, fat diet group; RFG, fat diet with resveratrol; ALT, alanine aminotransferase; AST, aspartate aminotransferase; CREA, creatinine; TG, triglycerides; TC, total cholesterol; HDL-C, high-density lipoprotein cholesterol; LDL-C, low-density lipoprotein cholesterol; Lp-PLA2, lipoprotein-associated phospholipase A2.

**Table 4:** Pathological parameters of aortic arch atherosclerosis observed under the microscope of RFG and FDG.

	Group	Mean	SD (±)	*P*
Intima	FDG	124.76	6.83	0.001
	RFG	52.44	14.94	

Smooth muscle layer	FDG	194.16	10.2	0.65
	RFG	173.48	4.05	

Intima/media ratio	FDG	0.64	0.04	0.001
	RFG	0.30	0.09	

Unit, *μ*m; SD, standard deviation; FDG, fat diet group; RFG, fat diet with resveratrol.

## Data Availability

The data used to support the findings of this study are included within the article.

## Additional Points

*Highlights*. (1) Resveratrol reduced the thickness of the aortic intima and the ratio of intima/media. (2) Lp-PLA2 levels were significantly reduced by resveratrol. (3) TC, LDL-C, and CREA levels were significantly different in RFG and FDG.
